# Comparison between the pharmacokinetics of meranzin hydrate in a rat model of chronic depression and in controls following the oral administration of Chaihu-Shugan-San

**DOI:** 10.3892/etm.2013.1229

**Published:** 2013-07-23

**Authors:** WEIBIN XIE, XINJIAN QIU, XI HUANG, YING XIE, KAIGE WU, YANG WANG, HUI JI, JUAN HE, PING REN

**Affiliations:** Department of Integrative Medicine of Traditional Chinese Medicine and Western Medicine, Xiangya Hospital, Central South University, Changsha, Hunan 410008, P.R. China

**Keywords:** chronic mild stress, meranzin hydrate, pharmacokinetics, Chaihu-Shugan-San

## Abstract

Previous studies have shown that meranzin hydrate (MH) may be beneficial in depressive disorders. However, to the best of our knowledge, the pharmacokinetic characteristics of MH in depression have not previously been investigated. Chronic mild stress (CMS) in rats is used as a model of depression. The present study was designed to evaluate and compare the pharmacokinetics of MH in CMS and control rats following the oral administration of Chaihu-Shugan-San (CSS). Rats were randomly divided into CMS and control groups and blood samples were obtained following the oral administration of CSS. The quantification of MH levels in the plasma for pharmacokinetic study was achieved using a simple and rapid ultra-performance liquid chromatography with photodiode array (UPLC-PDA) method. Following the oral administration of CSS to CMS rats and controls, the maximum plasma concentration (C_max_) of MH was 58.66±6.64 and 57.54±12.67 ng/ml at 108.00±26.83 and 54.00±8.22 min, respectively. Compared with the value of the area under the concentration-time curve (AUC)_0-1440_ in control rats (19,896.76±1,041.95 μg·min/l), the AUC_0-1440_ value was reduced in CMS rats (18,401.32±4332.65 μg·min/l). There were no significant differences in the majority of the pharmacokinetic parameters of MH, including the values for C_max_, AUC_0-1440_, clearance rate (CL/F) and mean residence time (MRT_0-1440_), between the CMS rats and the controls. However, the pharmacokinetic parameters showed that CMS accelerated the absorption of MH in rats following the oral administration of CSS.

## Introduction

Depression is a psychiatric disorder that presents as a reduction in self-confidence and a lack of confidence in the world and the future (1). Its core symptoms involve a depressed mood, anhedonia, irritability, difficulties in concentrating and abnormalities in appetite and sleep (2). Interestingly, Gan-Zhu-Shuxie (GZSX) theory, derived from traditional Chinese medicine (TCM), has been implicated in mood, digestion and reproduction (3). The dysfunction of GZSX may result in depression and a series of comorbidities, such as functional gastrointestinal and reproductive disorders.

Chaihu-Shugan-San (CSS) has been demonstrated to act as an antidepressant with polypharmacological mechanisms (4). CSS has been used for several centuries to improve certain symptoms similar to depression (5). Importantly, we previously isolated meranzin hydrate (MH) from CSS by targeting the unknown absorbed compound that was found in the blood of patients with depression following oral CSS administration (6). Furthermore, we isolated MH from Fructus Aurantii (FA) for the first time (Xi H, Ping R and Feng Q: Application and preparation of bitter orange extract meranzin hydrate for preparing enterocinetic kinetic edicaments. Filed January 8th 2008; issued January December 1, 2011), and conducted further studies with the compound (7,8).

To the best of our knowledge, there have been no studies simultaneously investigating the pharmacokinetic parameters of MH in a rat model of depression and in healthy rats following CSS administration. Chronic mild stress (CMS) in rats is a model of depression. The aim of this study was to evaluate and compare the pharmacokinetics of MH in the two types of rats.

A rapid, sensitive, simple and accurate ultra-performance liquid chromatography with photodiode array (UPLC-PDA) method to determine MH levels in the plasma of CMS rats and controls was developed and successfully applied in this pharmacokinetic study.

## Materials and methods

### Crude drugs

The CSS decoction included seven crude drugs: Bupleurum root, Pericarpium Citri Reticulatae, Chuanxiong Rhizoma, Rhizoma Cyperi, Fructus Aurantii, Paeonia and Radix Glycyrrhizae in a ratio of 8:5:5:5:5:3:2. All the dry herbs were purchased from Xiangya Hospital, Central South University (Changsha, China) and identified by the directing pharmacist, Xinzhong Li (Xiangya Hospital, Central South University). The voucher specimen (no. 20120910) was deposited in the laboratory of ethnopharmacology (Xiangya Hospital), prior to the herbs being immersed in distilled water (1:8, g/ml) for 1 h and boiled twice for 30 min. The blended supernatants were lyophilized to obtain a powdered form of CSS, which then was stored at 4°C until use.

### Chemicals and reagents

**Figure 1.**
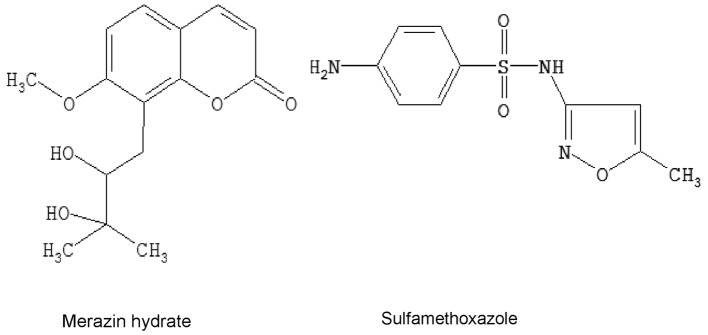
The chemical structures of meranzin hydrate and sulfamethoxazole.

MH was purchased from the Huaxi Medical University Medicine Factory (Chengdu, China), while sulfamethoxazole (SMZ; CAS No. 723-46-6, USP 98%) was provided by the National Institute for Food and Drug Control (Beijing, China) and was used as an internal standard (IS). The chemical structures of MH and SMZ are shown in Fig. 1. Acetonitrile and methanol [high-performance liquid chromatography (HPLC) grade] were obtained from Tedia Co., Inc. (Fairfield, OH, USA). All other reagents were of analytical grade. House triple-distilled water from silica glass equipment was always used.

### Chromatographic conditions

The Acquity UPLC system consisted of a binary solvent manager, a sample manager, a column heater and a PDA detector and was acquired from Waters Corp. (Milford, MA, USA). The PDA optical detector was an ultraviolet spectrophotometer that operated between 190 and 500 nm and the analytical column was a Waters BEH C18 column (2.1×100 mm i.d.) with a particle size of 1.7 *μ*m. The mobile phase comprised acetonitrile (A) and 0.5% aqueous acetic acid (B) with a gradient mode of 0–1 min, 3–3% A; 1–3 min, 3–15% A; 3–5 min, 15–18% A; 5–7 min, 18–20% A and 7–9 min, 20–30% A, v/v. The column temperature was maintained at 40°C and the autosampler was conditioned at 25°C. The flow rate was 0.4 ml/min and the injection volume was 6 *μ*l.

### Determination of MH in the CSS decoction

The lyophilized powder of CSS was dissolved in distilled water and an aliquot (1.0 ml) of the solution was extracted with methanol (9.0 ml). The extract solution was vortexed for 3 min and subsequently centrifuged for 10 min at 12,000 × g. The supernatant solution was filtered through a 0.22 *μ*M filter unit (EMD Millipore Corporation, Billerica, MA, USA), prior to UPLC analysis. The content of MH in CSS was measured under the aforementioned chromatographic conditions.

### Animals

Experiments were performed using male Sprague Dawley rats (weight, 200–220 g) provided by the Animal Experimental Center in Kaifu District (Changsha, China). All experiments conformed to the Regulations for the Administration of Affairs Concerning Experimental Animals (1988), and were approved by the Animal Experimental Center for Central South University. Animals were housed in a temperature-controlled facility with a 12-h light/dark cycle. The animals were acclimatized to the facilities for one week and were then randomly divided into two groups: i) the healthy control group (n=8) and ii) the CMS model group (n=7).

### CMS procedure

The CMS stressors were adapted from the procedure described by First *et al* (9) and the procedure consisted of a variety of unpredictable mild stressors, including: two periods (18 h) of grouped caging, with four rats per cage (usually CMS rats were housed like the controls, one per cage); two periods (6 h) of cage tilting (tilted by 30° on a wooden board); one period (24 h) of food deprivation; two periods (24 and 46 h) of water deprivation; one period (18 h) of a wet cage (200 ml water spilled in each cage); two periods (3 h) of stroboscopic lightning (a flashlight flickering at 300 flashes/min in a dark room); two periods (3 h) of white noise (a non-tuned radio on high volume) and one 48-h period of continuous light. These stressors were randomly scheduled over a one-week period and repeated throughout the five-week procedure.

The non-stressed control animals were housed in constant conditions, with one rat per cage, i.e. similar to the stressed animals, but without any manipulations. We were aware of the fact that social isolation by itself is a stressor for rodents, who are naturally social animals; however, we overcame this limitation by exposing all groups to the same housing conditions.

### Open field test

The open field test was performed in accordance with a previous study (10), in order to measure spontaneous activity in rodents. Briefly, the apparatus, consisting of a black square cage measuring 100×100×40 cm, was divided into 25×25 cm equal squares on the floor of the arena. The test room was dimly illuminated. A single rat was placed in the center of the cage and, following 30 sec of adaptation, the crossing number (CN, i.e. a rat stepping from one square to another with its rear legs) was utilized as the measurement parameter. A separate researcher, who was blind to the treatment group, scored the behavior in the open field. Following each test the arena was cleaned with 90% alcohol solution.

### Forced swimming test

In accordance with the study by Porsolt *et al* (11), the forced swimming test was conducted by placing the rat in a Plexiglas^®^ cylinder (40 cm tall, 30 cm in diameter) filled to a height of 21.5±1.5 cm with water at a temperature of 24±0.5°C. The rats were left to swim in the cylinder under conditions where escape was not possible. To avoid additional stress on the animals, the original forced swimming test described by Porsolt *et al* (11) was modified by performing a single test session lasting for 5 min, during which the animals were videotaped from a vantage point above the cylinders in a dimly illuminated room. The duration of immobility, which was defined as the lack of motion of the whole body, except for small movement necessary to keep the animal’s head above the water, was recorded. Subsequent to each test, the cylinder was cleaned.

### Preparation of standard and quality control (QC) samples

Standard stock solutions were prepared by dissolving MH and SMZ in methanol to yield nominal concentrations of 64 *μ*g/ml and 100 *μ*g/ml, respectively, for storage at 4°C. The solutions were subsequently further diluted in methanol to produce working standards. Calibration samples of MH (2.5, 10, 40, 80, 160, 320 and 640 ng/ml) were prepared by spiking 1 ml blank plasma with appropriate quantities of working standard solutions and SMZ (IS, 50 *μ*l). QC samples of MH were independently prepared at three different concentration levels (10, 40 and 160 ng/ml) to determine the recovery, accuracy and the precision of the method. All the plasma samples were stored at −20°C, prior to analysis.

### Plasma sample preparation

The blank plasma (1 ml) in a centrifuge tube (5 ml) was added to different amounts of MH (2.5–640 ng, 50 *μ*l), a fixed quantity of SMZ (5,000 ng, 50 *μ*l) and 850 *μ*l methanol. The mixture was mixed thoroughly by ultrasound and vortexed for 30 sec. Following this, the denatured protein precipitate was separated by centrifugation at 3,000 × g for 15 min at 4°C and the supernatant was transferred to another tube and evaporated to dryness in a water bath at 50°C under a stream of nitrogen. The residues were reconstituted in methanol (50 *μ*l), prior to being vortexed for 15 sec. The centrifugation procedure was repeated as mentioned previously and then 6.0 *μ*l supernatant solution was injected into the UPLC system for analysis.

### Calibration curve and limit of quantification (LOQ)

Standard samples of MH (2.5–640 ng/ml) and 5,000 ng SMZ (IS) in plasma were prepared as previously mentioned. Standard curves were established following the extraction and UPLC analyses of the spiked plasma samples. Following the determination of the peak-area ratios of MH to SMZ in the UPLC chromatograms, the calibration curve was established by least-squares linear fitting of the peak-area ratios of MH to the IS. The LOQ was defined as the lowest concentration.

### Precision and accuracy

The intra-day accuracy and precision were assessed by determining QC samples at three concentration levels of MH (10, 40 and 160 ng/ml) on the same day (n=6). The inter-day accuracy and precision were also evaluated from the analysis of the QC samples on three consecutive days (n=6). Precision was expressed as relative standard deviation (RSD) and accuracy was expressed as [(mean detected concentration-added concentration)/(added concentration)] x 100.

### Recovery

The relative recoveries of MH from rat plasma were determined using the QC samples (n=6). The peak-area ratios (MH to SMZ) of the UPLC chromatograms were compared with those of reference solutions to calculate the relative recoveries of MH.

### Stability in rat plasma

The short-term, long-term and freeze-thaw stabilities of MH in plasma were assessed using QC samples (n=6). Short-term stability was assessed by analyzing QC plasma samples kept at room temperature for 4 h, which exceeded the routine preparation time of the samples. Long-term stability was determined by assaying QC plasma samples following storage at −20°C for 14 days. Freeze-thaw cycles (−20°C/room temperature) were also applied to QC samples to investigate the freeze-thaw stability of MH. In each freeze-thaw cycle, the samples were frozen and stored at −20°C for 24 h, and subsequently thawed at room temperature.

### Pharmacokinetic study

Following the behavior tests, the two groups were fasted with free access to water for 12 h prior to the experiment. CSS was orally administered to the rats at a dose of 30 g/kg (for raw medicinal materials). Blood samples (0.3 ml) were collected in heparinized tubes at 0 (prior to administration), 5, 10, 15, 45, 60, 120, 240, 300, 360, 480, 720 and 1,440 min subsequent to administration, left for 30 min at room temperature and then centrifuged at 12,000 × g for 10 min at 4°C, in order to obtain the plasma. The supernatant was transferred and stored in 0.5-ml polypropylene tubes at −20°C prior to analysis. The next step was performed following the plasma sample preparation. The concentrations of MH in the rat plasma were determined at each time-point. Data from those samples were used to construct the pharmaco-kinetic profiles by plotting drug concentration versus time curves. Pharmacokinetic parameters, including area under the concentration-time curve (AUC_(0-t)_), maximum plasma concentration (C_max_), time to reach the maximum concentration (T_max_), clearance rate (CL/F) and mean residence time (MRT_(0-t)_), were estimated using statistical moment analysis with Drug and Statistics 2.0 (DAS 2.0) software (Mathematical Pharmacology Professional Committee of China, Shanghai, China).

### Statistical analysis

All data are expressed as the mean ± standard deviation. The database was set up with the SPSS 16.0 software package from SPSS, Inc. (Chicago, IL, USA). Differences between the two groups were analyzed using one-way analysis of variance. P<0.05 was considered to indicate a statistically significant difference.

## Results

**Figure 2.**
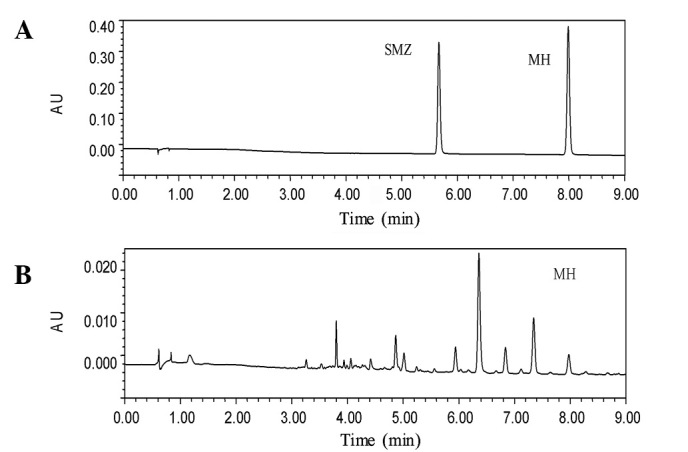
Typical chromatograms of standards and the Chaihu-Shugan-San CSS test sample. (A) The first peak is the internal standard and the second peak is meranzin hydrate (MH); (B) CSS test sample. SMZ, sulfamethoxazole.

**Figure 3.**
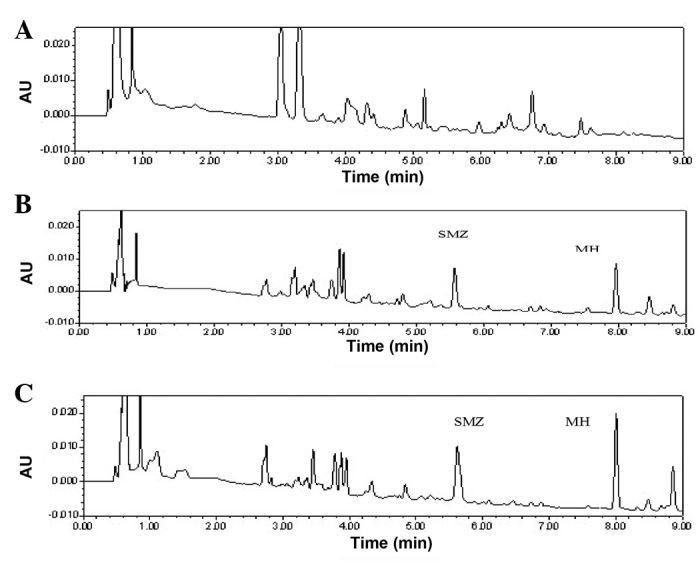
Chromatograms of (A) blank plasma sample; (B) plasma sample spiked with meranzin hydrate (MH) and sulfamethoxazole (SMZ; internal standard); (C) plasma sample of a rat with chronic mild stress (CMS), obtained 60 min subsequent to oral administration of Chaihu-Shugan-San (CSS).

The content of MH in the CSS decoction was calculated to be 113.16 *μ*g/g. Typical chromatograms of authentic standards and the CSS test sample are shown in Fig. 2, while representative chromatograms of a blank control sample, spiked plasmas and a subject sample are depicted in Fig. 3. MH and SMZ in the plasma were completely separated, without significant interference. The retention times of MH and SMZ were 7.9 and 5.6 min, respectively, and it was observed that the calibration curve was linear over the concentration range of 2.5–640 ng/ml in the rat plasma. The representative regression equation of the calibration curve was y=315.3 and x=−2.589 (r^2^=0.9995, n=6) and the LOQ and limit of detection (LOD) were 3 and 1 ng/ml, respectively. The precision and accuracy of the method are summarized in Table I. The intra- and inter-day precisions were ≤1.63 and ≤1.54%, respectively, and the mean recovery ratios of MH at concentrations of 10, 40 and 160 ng/ml were demonstrated to be 94.25±1.69, 95.67±1.57 and 97.76±1.37%, respectively with all RSDs ≤1.79% (Table II). Table III summarizes the results of the short-term, long-term and freeze-thaw stability of MH in rat plasma. The stability of MH in rat plasma was acceptable in the present study.

**Figure 4.**
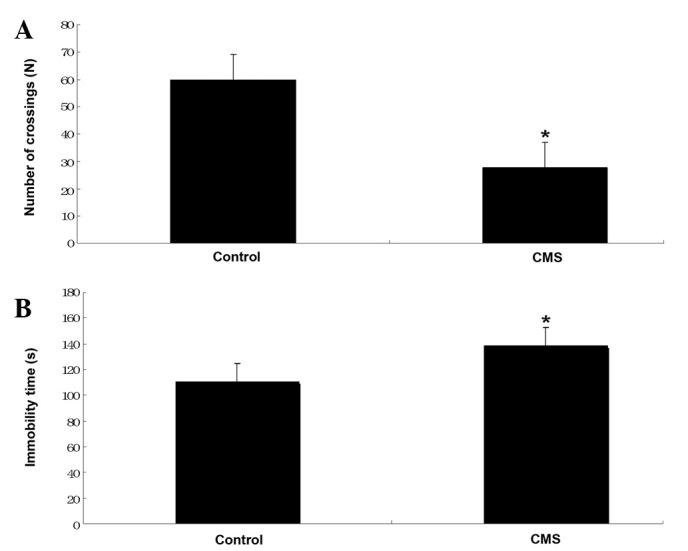
(A) Number of crossings in the open field test. ^*^P<0.05 vs. control; (B) Immobility time in the forced swimming tests. ^*^P<0.05 vs. control. CMS, chronic mild stress.

The immobility time (sec) and the number of crossings (n) observed for the rats with CMS were compared with the values obtained for the control rats in Fig. 4. The immobility time was shown to increase significantly in the CMS rats (138.4±11.4 versus 110.5±7.2 sec for the control), while the number of crossings was reduced (27.7±11.0 versus 59.8±26.4 for the control). These data indicated the success of the model of chronic depression.

**Figure 5.**
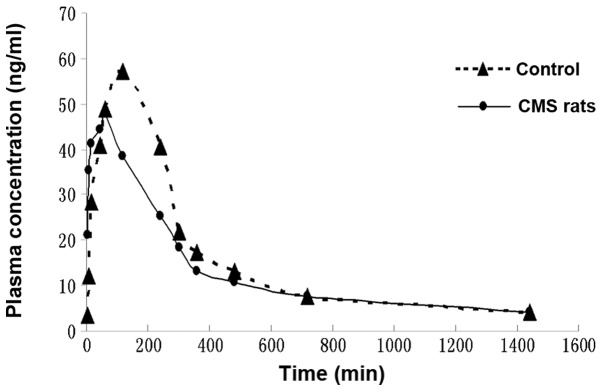
Plasma concentration-time profiles of meranzin hydrate (MH) in rats with chronic mild stress (CMS) and controls following the oral administration of Chaihu-Shugan-San (CSS; 30 g/kg).

The plasma MH concentration-time curves were analyzed using DAS software (Mathematical Pharmacology Professional Committee of China) on a personal computer to determine the compartment model, and the plasma concentration-time curve of MH was fitted with a two-compartment model. The mean plasma concentration versus time profiles of MH in the CMS rats and the controls, following the oral administration of CSS, are illustrated in Fig. 5. The main pharmacokinetic parameters, including the T_max_, C_max_, half-life (T_1/2_), K_a_, AUC_0-1440_, MRT_0-1440_ and CL/F were calculated for each group and are listed in Table IV.

Following the oral administration of CSS to the control rats, MH was absorbed and reached a C_max_ value of 58.66±6.64 *μ*g/l within 108.00±26.83 min. The plasma concentration of MH declined with a T_1/2_ value of 87.34±31.15 min. However, following the oral administration of CSS to the CMS rats, the C_max_ value of MH was 57.54±12.67 *μ*g/l within 54.00±8.22 min, and the plasma concentration of MH declined with a T_1/2_ value of 145.64±75.67 min.

## Discussion

Previous studies have implicated the usage of MH in anti-cancer (12), antibacterial and anticoagulation (13) treatments. However, we have identified additional effects exhibited by MH. *In vivo,* MH (28 mg/kg) significantly accelerated gastric emptying and intestinal transit in rats, and also directly increased the amplitude of rat ileum contraction *in vitro* (8). Furthermore, *in vivo* MH (1–100 *μ*M) dose-dependently increased the amplitude of contractility in the longitudinal and circular jejunum muscles of rats, and this was, at least partially, mediated by the stimulation of H1 histamine receptors (14). Therefore, MH was selected as the target to compare the pharmacokinetic profiles in CMS rats and control rats.

The pharmacokinetic profile of MH in rat plasma was fitted with a two-compartment model, detected by a simple, rapid and accurate UPLC method. SMZ was selected as the appropriate IS, as it was stable and did not exist in rat blank control plasma. The total analysis time was 9.0 min and the retention time of MH was 7.9 min, which was significantly shorter than that achieved in a previous method, with better resolution (6–8).

The acceptable peak shape and satisfactory separation of MH and SMZ from endogenous components were achieved in rat plasma under the previously mentioned chromatographic conditions. The method was validated for linearity, accuracy, precision, LOQ and recovery and was successfully applied to the pharmacokinetic study of MH in CMS rats and controls.

The pharmacokinetic parameters determined in the current study showed that CMS accelerated the absorption of MH in rats following oral administration of CSS. There were differences between our study and others. A previous study investigated the potential effect of MH and a decoction of the herb, FA, on rat gut motility, in addition to investigating the prokinetic mechanism of MH (14). The study utilized normal rats, not CMS rats, in contrast to the present study (14). Another study aimed to identify an antidepressive compound found in TCM by a novel approach known as ethnopharmaco-kinetic- and activity-guided isolation (EAGI) (6). In this study, MH, a compound whose antidepressive effect is similar to FA and CSS, was isolated for the first time from FA (6). This was achieved by targeting its corresponding unknown chromatographic peak, and its antidepressive effect was compared with FA or CSS (6). Similar to the present study, UPLC was used, as a development of the chromatographic technique, in the quality control of the TCM, CSS. However, the study did not describe the pharmacokinetic parameters of MH in a rat model of depression, in addition to healthy rats, following CSS administration (6). Thus, to the best of our knowledge, the present study has shown for the first time that CMS accelerated the absorption of MH in rats following the oral administration of CSS.

Our experiments have compared the pharmacokinetics of MH in a rat model of chronic depression and control rats following the oral administration of CSS. CSS is one of many traditional Chinese antidepressant drugs. The Chinese herbal formula, Mood Smooth (Jia Wei Xiao Yao Wan), has been in use for six hundred years in China as a treatment for depression. The Chinese refer to this remedy as ‘the happy pill’, due to its well-known antidepressant effect. It has been used by millions of people over the centuries, and is particularly popular with females. Other common remedies for depression include: Spleen tonic herbal formula, Chi Spleen Tonic (Bu Zhong Yi Qi Wan); kidney nourishing herbal formula, Kidney Yang Tonic (Jin Gui Shen Qi Wan) and numerous other remedies that are widely used for different patterns of depression. Further studies to investigate the pharmacokinetic effects of other orally administered traditional Chinese antidepressant drugs in rat models of chronic depression and control rats are required.

## Figures and Tables

**Table I.** Precision and accuracy of the UPLC method for MH in rat plasma (n=6).

Nominal concentration (ng/ml)	Intra day (n=6)		Inter day (n=6)	
	
	Mean ± SD (ng/ml)	RSD (%)	Mean ± SD (ng/ml)	RSD (%)
10	9.67±0.13	1.34	9.87±0.12	1.22
40	39.21±0.52	1.33	39.01±0.60	1.54
160	153.07±2.50	1.63	150.72±1.53	1.01

UPLC, ultra-performance liquid chromatography; MH, meranzin hydrate; RSD, relative standard deviation.

**Table II.** Recovery of MH from rat plasma.

Concentration (ng/ml)	Recovery (%) (mean ± SD)	RSD (%)
10	94.25±1.69	1.79
40	95.67±1.57	1.64
160	97.76±1.37	1.40

MH, meranzin hydrate; RSD, relative standard deviation.

**Table III.** Stability of MH in rat plasma at three QC levels (n=6).

Stability	Nominal concentration MH (ng/ml)		
	10	40	160
Short term stability	9.49±0.61	38.61±0.70	147.43±3.05
Long term stability	9.28±0.75	38.44±0.65	147.25±2.21
Freeze thaw stability	9.18±0.47	38.35±0.59	146.63±2.06

QC, quality control; MH, meranzin hydrate.

**Table IV.** Pharmacokinetic parameters of MH in plasma following the oral administration of CSS to CMS and control rats.

Parameter	CMS rats	Control rats
T_max_ (min)	54.000±8.216^a^	108.000±26.830
C_max_ (*μ*g/l)	57.544±12.673	58.664±6.640
AUC_0-1440_ (*μ*g·min/l)	18401.317±4332.648	19896.758±1041.950
T_1/2_ (min)	145.635±75.671	87.338±31.145
K_a_ (1/min)	0.083±0.074	0.021±0.009
CL/F (l/min/kg)	1442.188±391.815	1445.447±77.808
MRT_0-1440_ (min)	409.953±43.412	378.751±13.028

Values are presented as the mean ± standard deviation. n=7 in chronic mild stress (CMS) rats; n=8 in control rats;

a P<0.05 compared with control rats. MH, meranzin hydrate; CSS, Chaihu-Shugan-San; T_max_, the time to reach peak concentration; C_max_, maximum plasma concentration; AUC, area under the concentration-time curve; T_1/2_, apparent elimination half-life; K_a_, absorption constant; CL/F, apparent clearance; MRT, mean residence time.
